# Atypical Case Presentation of Toxic Shock Syndrome

**DOI:** 10.7759/cureus.44429

**Published:** 2023-08-31

**Authors:** Folake Ishola, Gurvir Kaur Mangat, Kayla Martinez, Yaqub Nadeem Mohammed, Malik McKany

**Affiliations:** 1 Internal Medicine, Ross University School of Medicine, Pontiac, USA; 2 Surgery, Ross University School of Medicine, Pontiac, USA; 3 Internal Medicine, Trinity Health Oakland Hospital, Pontiac, USA; 4 Surgery, Trinity Health Oakland Hospital, Pontiac, USA

**Keywords:** sepsis-associated dic, rhabdomyolysis, septic shock, streptococcal toxic shock syndrome, bacteremia, staphylococcus aureus, group a streptococcus, streptococcus pyogenes, toxic shock syndrome (tss)

## Abstract

Toxic shock syndrome is a rare and life-threatening condition that is typically caused by group A Streptococcus or Staphylococcus aureus. It classically presents with fever, hypotension, sunburn-like rash, and multi-organ system failure. We describe a case of a 70-year-old male with this condition who had an atypical presentation of left chest wall pain and left shoulder pain after two mechanical falls along with hemodynamic stability. The patient rapidly deteriorated on his second hospital floor day, resulting in a higher complexity of care and management in the intensive care unit (ICU). Despite a number of resuscitative measures, therapies, and multidisciplinary care, the patient unfortunately passed away within 24 hours of his ICU care.

## Introduction

Toxic shock syndrome is a critical condition that is typically caused by exotoxin-producing group A Streptococcus (S. pyogenes) and Staphylococcus aureus [1]. It usually presents with fever, hypotension, sunburn-like rash, and multiorgan system failure [1,2]. This condition is known to be linked with ultra-absorbent tampon use in menstruating women, resulting in the use of a single tampon for a longer duration [1]. However, other remarkable sources of non-menstrual toxic shock syndrome are burns, surgical wounds, nasal packing, dialysis catheters, trauma, and soft tissue infections [1,3]. In some cases, the source of the toxic shock syndrome is not found during workup [3]. Staphylococcal toxic shock syndrome is commonly due to a focused source of infection such as an abscess, while streptococcal toxic shock syndrome is mostly due to conditions such as bacteremia and necrotizing fasciitis [1]. In the United States, the incidence rate of toxic shock syndrome is about 0.8-3.4% per 100,000 persons [1]. The occurrence of this condition tends to peak in the winter season and is commonly seen in developing countries [1]. Patients with staphylococcal toxic shock syndrome have a mortality rate of less than 3% [1]. Individuals with streptococcal toxic shock syndrome have a mortality rate from 23% to 44% [4]. We report a case of streptococcal toxic shock syndrome with an atypical presentation of left chest wall pain and left shoulder pain after two mechanical fall episodes, as well as the rapid decompensation of the patient in 24 hours.

## Case presentation

**Figure 1:**
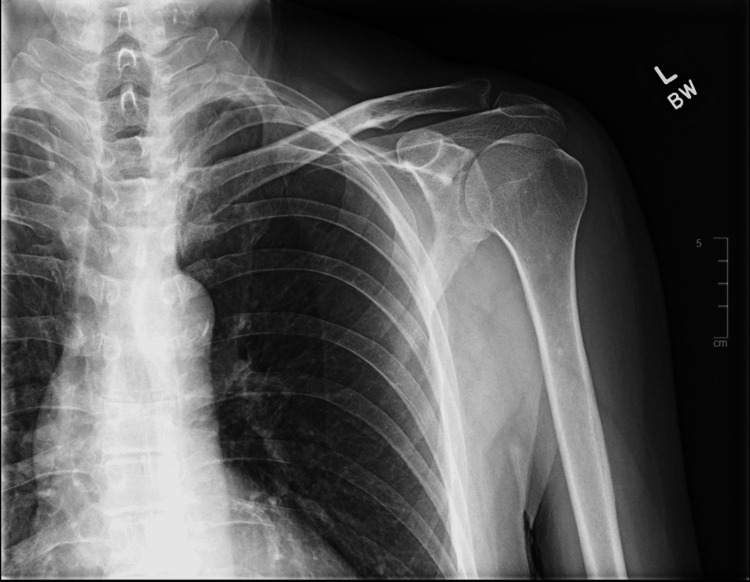
X-ray of the left shoulder No acute fracture or posttraumatic left shoulder dislocation. Acromioclavicular and coracoclavicular intervals are maintained. Mild osteoarthrosis of the left shoulder with spurring about the acromioclavicular joint.

A 70-year-old Caucasian male, with a past medical history of hypertension, hyperlipidemia, coronary artery disease s/p angioplasty in 2019, and prostate cancer in remission s/p prostatectomy in 2013, presented to the Emergency Department (ED) for left chest wall pain and left shoulder pain after two different mechanical falls. The mechanical fall episodes occurred three and two days prior to ED presentation. Chest X-ray, left shoulder X-ray, and thoracic spine X-ray all showed no acute findings, with no fracture or traumatic malalignment noted. Figure 1 shows a plain film of the left shoulder. He was discharged on Flexeril and lidocaine patches after reevaluation for symptomatic relief.

**Figure 2:**
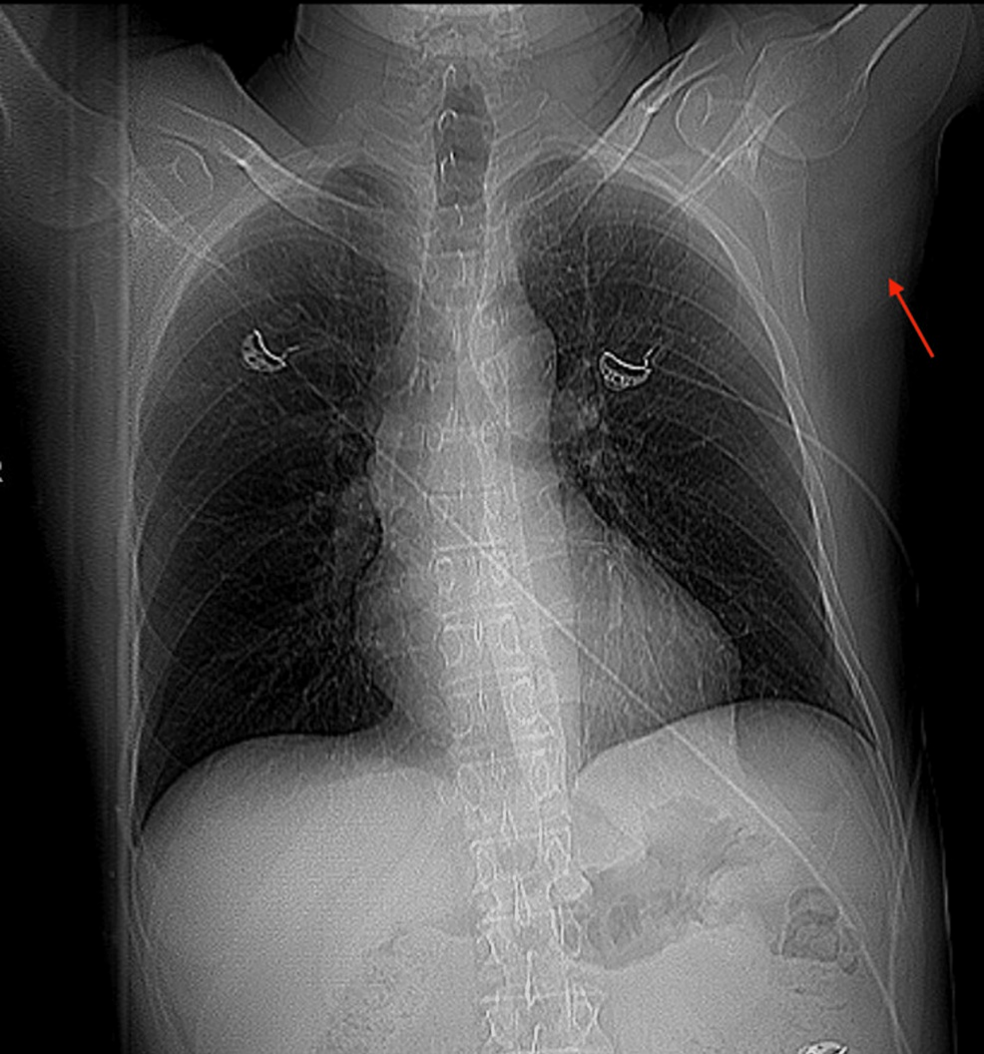
CT of the chest Nonspecific heterogenous attenuation/soft tissue edema lateral to the left pectoralis major with a few internal nonenlarged prominent lymph nodes with obscuration of the left anterior subscapularis muscle, as indicated by the red arrow.

**Figure 3:**
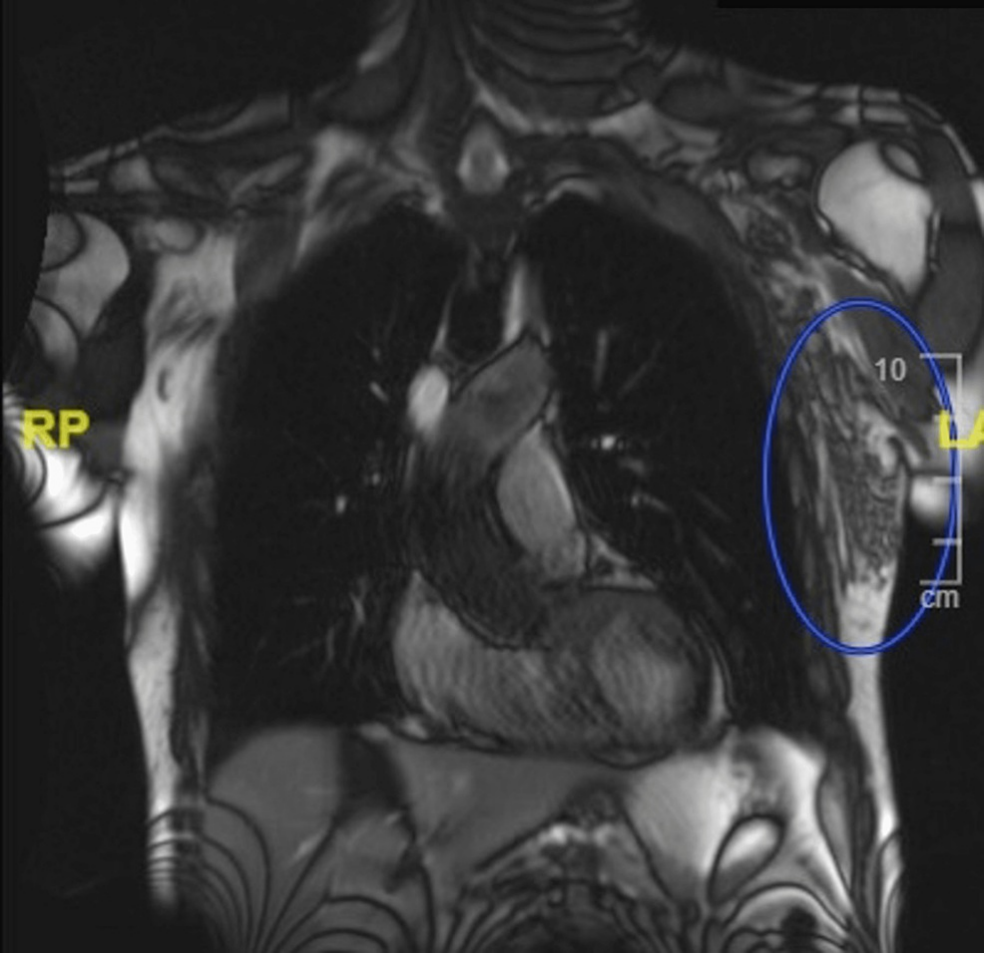
MRI of the chest Posttraumatic contusion in the left axilla with straining of the left latissimus dorsi, left subscapularis, left triceps, and biceps musculature. More confluent edema signal within the substance of the left latissimus dorsi and subscapularis muscles, suggestive of muscle tearing and intramuscular hematoma, as indicated by the blue circle. No acute fracture.

Following the discharge, the patient presented again to the ED later that day for severe pain in the left lateral chest wall that was aggravated by left arm movement. CT chest without contrast revealed a nonspecific area of attenuation with soft tissue edema lateral to the left pectoralis major with a few internal nonenlarged lymph nodes that obscure the left subscapularis muscle (Figure 2). The patient was admitted to the floor for IV pain medication management and MRI chest imaging to rule out malignancy based on the presence of lymph nodes on CT imaging. MRI chest with and without contrast showed a 5.4 x 2.2 cm posttraumatic contusion in the left axilla, with straining of the left latissimus dorsi, left subscapularis, left triceps, and biceps musculature (Figure 3). Edema within the substance of the left latissimus dorsi and subscapularis muscles was suggestive of muscle tearing and intramuscular hematoma. The MRI report showed no evidence of malignancy and the few enlarged lymph nodes were found to be nonspecific and likely reactive. No enlarged mediastinal lymph nodes were seen. Labs were unremarkable, except for a mildly elevated white blood cell count at 14.8.

**Figure 4:**
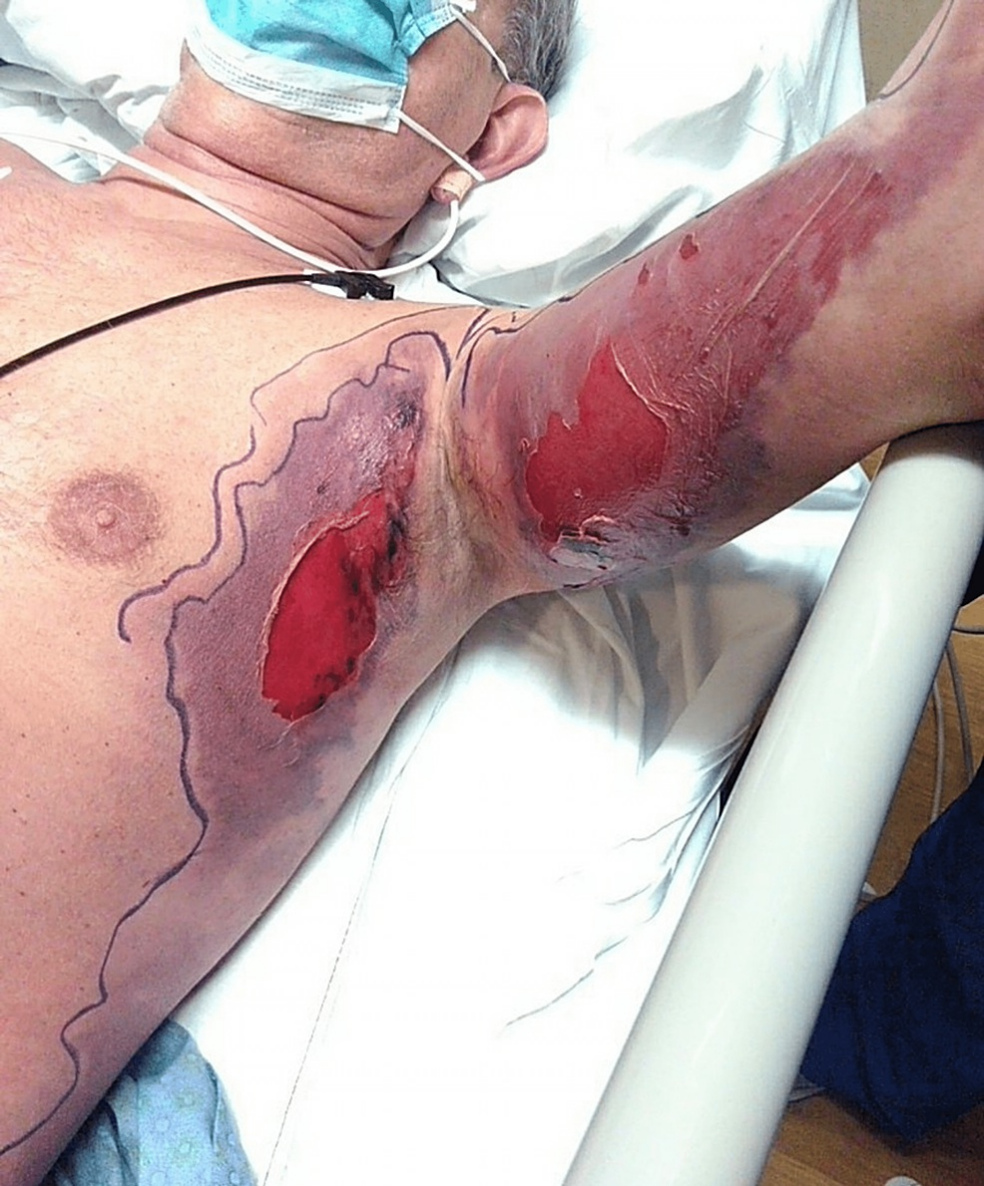
Left axilla and left arm, Intensive Care Unit Day 1 Patient's injury from mechanical fall episodes, taken on ICU Day 1.

The second hospital floor day rapidly turned into Intensive Care Unit (ICU) Day 1 as the rapid response team was called to the patient's room for altered mentation with confusion. STAT CT head showed no evidence of an acute intracranial abnormality or hemorrhage. CT left upper extremity (LUE) showed extensive edema and soft tissue swelling in the left axilla, extending from the left biceps into the thoracic chest wall along the left latissimus dorsi. The patient was started on aggressive IV resuscitation, clindamycin, vancomycin, and zosyn for possible necrotizing fasciitis. The physical exam of the patient at this time showed skin changes to the left axilla and left arm, as seen in Figure 4. Additionally, the patient's bilateral upper digit tips were cold to the touch and slightly purple in color. There was no evidence of compartment syndrome on the LUE arterial and venous duplex vascular ultrasound. Vascular surgery was consulted for possible drainage or debridement. They recommended ice packing and supportive management with no surgical intervention. The orthopedic surgery team saw the patient for the left latissimus dorsi muscle tear with associated hematoma and recommended no acute surgical intervention.

A few hours later, the patient decompensated rapidly becoming hypotensive, tachycardic, and lethargic with increased confusion. He was then transferred to the ICU for higher complexity of care. At this time, his lab work showed remarkable changes in elevated lactic acid, elevated liver function tests, elevated renal function tests, and elevated CK, as seen in the ICU Day 1 (PM) column in Table 1. Overnight, the patient became unconscious, alert and oriented, tachypneic, and anuric and was intubated, requiring four-pressor support. His condition became complicated with hyperkalemia, hypoglycemia, and severe lactic acidosis (as seen in ICU Day 2 (AM) in Table 1), along with elevated procalcitonin levels at 304.35. Overnight, he had a Quinton catheter placed with STAT continuous renal replacement therapy and bicarbonate drip. At this time, the blood cultures came back positive for S. pyogenes. General surgery was consulted for possible necrotizing fasciitis. MRI and CT imaging showed no evidence of necrotizing fasciitis, and the team suggested that the creatinine kinase levels were consistent with rhabdomyolysis.

**Table 1:** Hospital stay of remarkable laboratory values Progression in patient's laboratory values over his hospital stay.

	Hospital Floor Day 1	ICU Day 1 (AM)	ICU Day 1 (PM)	ICU Day 2 (AM)	ICU Day 2 (PM)	Reference Values
Hb	13.5 g/dL	14.0 g/dL	15.4 g/dL	10.1 g/dL	8.0 g/dL	13-18 g/dL
WBC	14.8 K/mcL	4.1 K/mcL	2.5 K/mcL	8.4 K/mcL	5.8 K/mcL	3.7-11.0 K/mcL
PLTs	192 K/mcL	160 K/mcL	153 K/mcL	61 K/mcL	40 K/mcL	150-450 K/mcL
K	3.9 mmol/L	4.5 mmol/L	5.2 mmol/L	7.6 mmol/L	7.9 mmol/L	3.5-5.3 mmol/L
pH arterial	-	-	-	7.21	7.13	7.35-7.45
Lactate	-	-	9.0 mmol/L	20.0 mmol/L	17.0 mmol/L	0.5-2.0 mmol/L
BUN	21 mg/dL	28 mg/dL	47 mg/dL	60 mg/dL	58 mg/dL	7-25 mg/dL
Creatinine	1.0 mg/dL	1.39 mg/dL	3.03 mg/dL	5.12 mg/dL	4.89 mg/dL	0.7-1.30 mg/dL
AST	-	-	63 unit/L	715 unit/L	1,424 unit/L	13-39 unit/L
ALT	-	-	28 unit/L	428 unit/L	1,028 unit/L	7-52 unit/L
ALP	-	-	31 unit/L	22 unit/L	57 unit/L	27-120 unit/L
Albumin	-	-	3.5 g/dL	1.6 g/dL	<1.5 g/dL	3.5-5.7 g/dL
Total Bilirubin	-	-	0.7 mg/dL	1.0 mg/dL	1.9 mg/dL	0.3-1.0 mg/dL
Total CK	-	-	2,756 unit/L	17,170 unit/L	>20,000 unit/L	50-280 unit/L
Prothrombin time	-	-	-	31.8 seconds	28.6 seconds	9.5-12.5 seconds
aPTT	-	-	-	73.0 seconds	56.9 seconds	26.0-36.0 seconds
INR	-	-	-	2.8	2.5	<6.0
Glucose	119 mg/dL	144 mg/dL	107 mg/dL	46 mg/dL	182 mg/dL	70-99 mg/dL
Calcium	9.3 mg/dL	8.6 mg/dL	7.7 mg/dL	5.9 mg/dL	5.5 mg/dL	8.6-10.3 mg/dL

**Figure 5:**
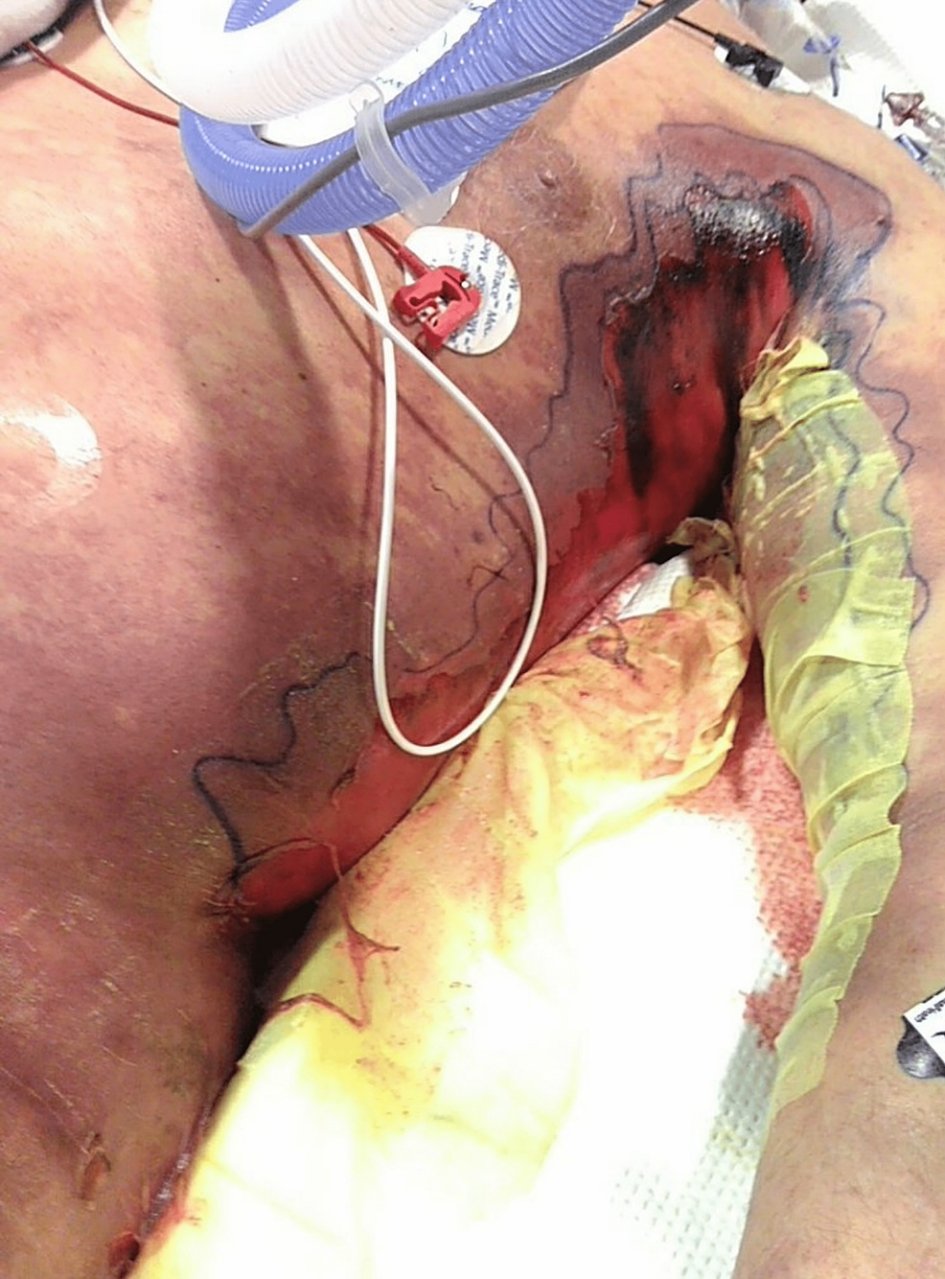
Left axilla and left arm, Intensive Care Unit Day 2 Worsening changes and area of necrosis on ICU Day 2, 24 hours after Figure 1 was taken.

**Figure 6:**
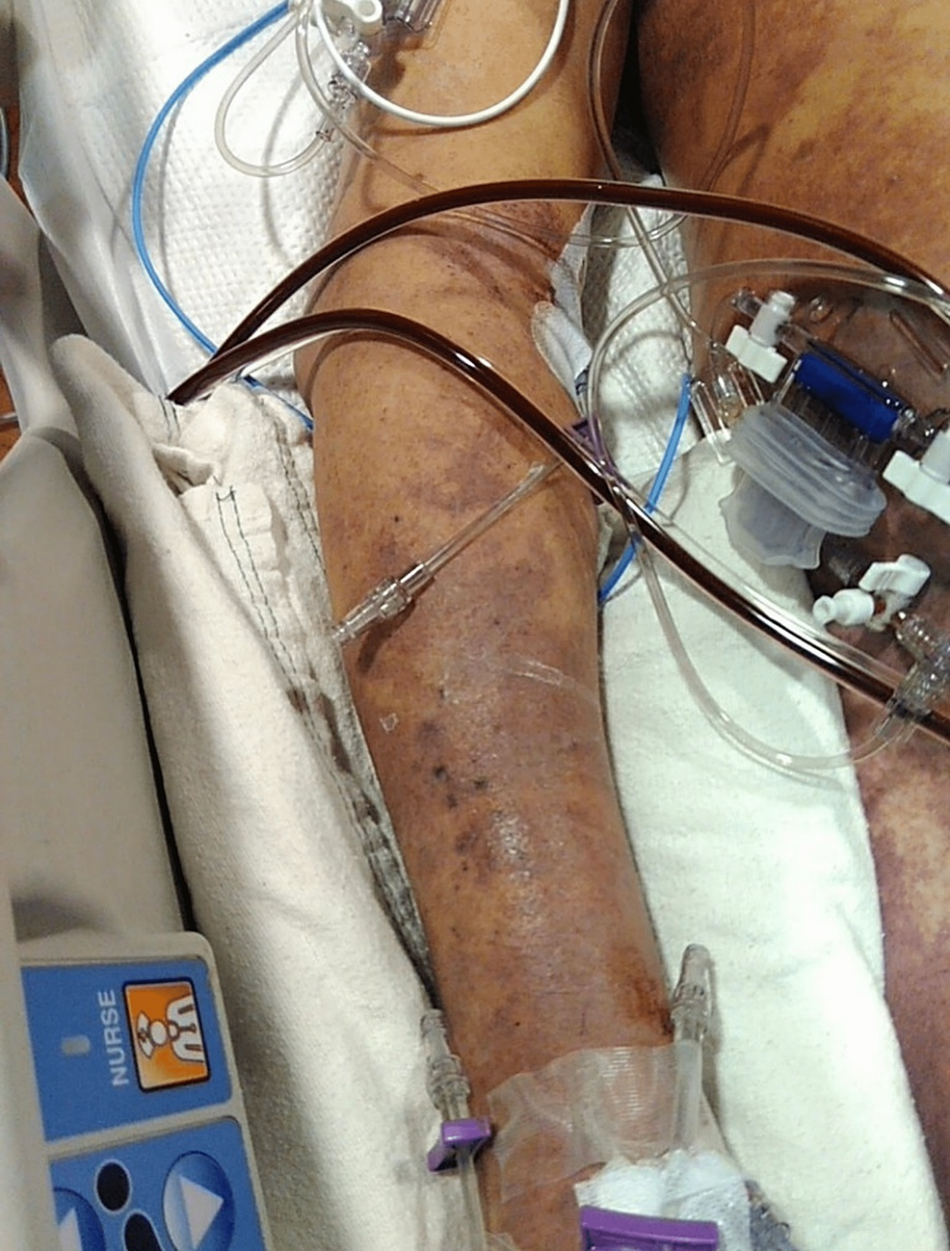
Right arm and right axilla, Intensive Care Unit Day 2 New skin changes to the patient's right arm and right axilla on ICU Day 2.

**Figure 7:**
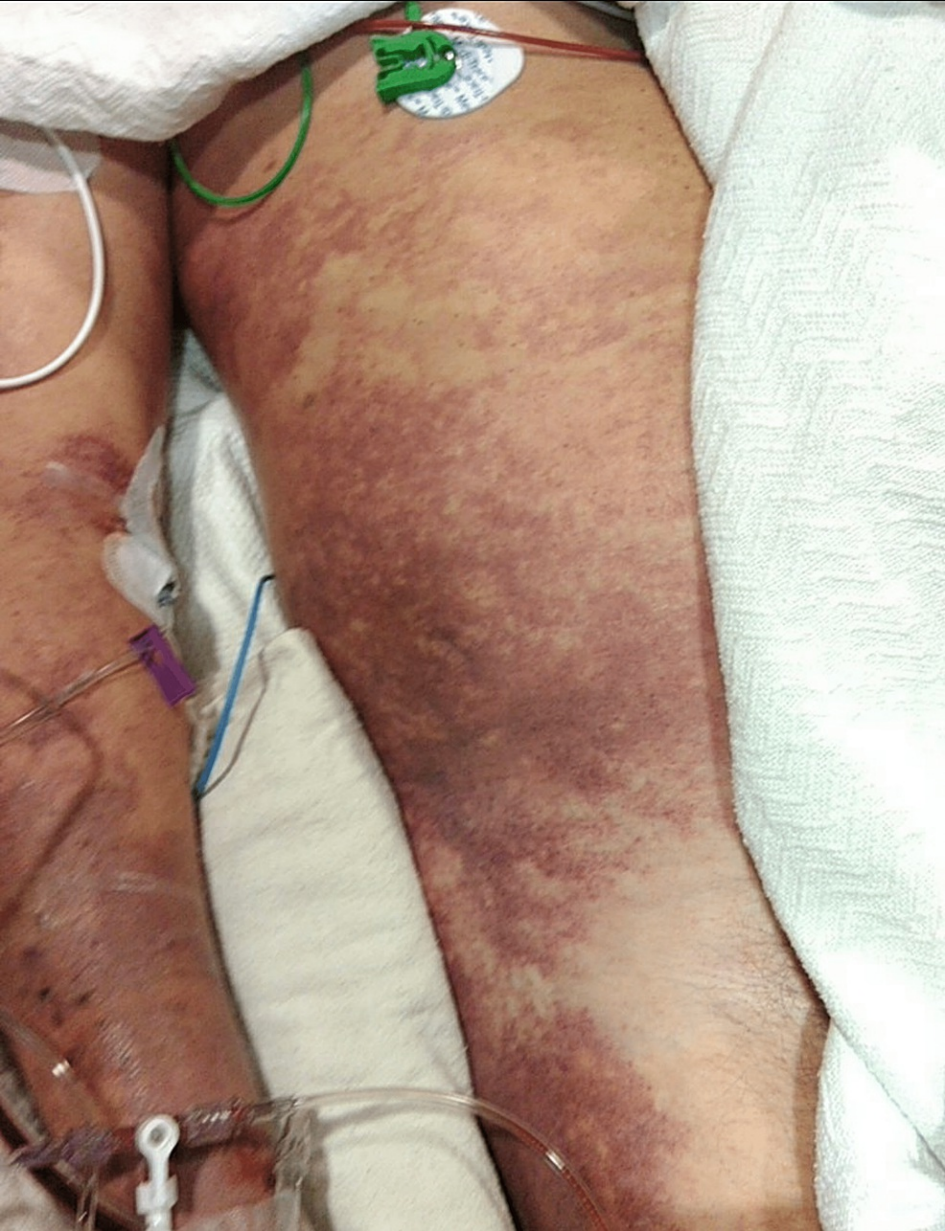
Bilateral legs, Intensive Care Unit Day 2 New skin changes and increased swelling to the patient's bilateral legs on ICU Day 2.

Due to the patient's rapid decompensation and the advanced skin changes of necrosis (as seen in Figure 5), he was evaluated by the palliative care team on ICU Day 2 (AM). Additionally, the patient had extensive swelling and mottling of the body (as seen in Figures 6-7), with multiorgan failure and rapid deterioration. Hematology was consulted for disseminated intravascular coagulation (DIC), and the patient was started on STAT 2 units of cryoprecipitate, 2 units of FFP, and a dose of vitamin K. At this time, the patient’s antibiotic regimen was switched to daptomycin, meropenem, and clindamycin, per the Infectious Disease team recommendations, and was started on intravenous immune globulin (IVIG). Despite the aggressive measures and multidisciplinary team care, the patient continued to rapidly decline and passed away in the evening of his ICU Day 2 stay.

## Discussion

In this condition, group A Streptococcus and S. aureus produce exotoxins with superantigen activity. Superantigens are known to cause abundant and widespread T-cell activation, resulting in the excessive secretion of pro-inflammatory cytokines and other factors causing capillary leak and arterial hypotension [5]. The involvement of superantigens in this disease process forms the basis of the systemic pro-inflammatory activity, arterial hypotension, and end-organ damage seen in affected individuals [6]. Toxic shock syndrome usually presents with fever, hypotension, sunburn-like rash, and multiorgan system failure [1,2]. Our patient had an atypical presentation of this condition, with left chest wall pain and left arm pain after two mechanical falls with hemodynamic stability. Due to this presentation at his first encounter in our ED facility, he was given symptomatic care and had imaging done. All the imaging studies revealed no acute or posttraumatic findings, resulting in his initial discharge with Flexeril and lidocaine patch.

The clinical presentation of toxic shock syndrome typically develops in 48 hours. Our patient decompensated on the second hospital floor day, which required escalation of care to the ICU, and passed away on his ICU Day 2. In the case of our patient, he developed the classic toxic shock presentation of hypotension and multiorgan failure within 24 hours. A study by Mehta et al. on the morbidity and mortality of 62 ICU patients with streptococcal toxic shock syndrome across four university hospitals in Toronto, Canada, showed that the median period for ICU and hospital stay were 5.3 and 15.0 days, respectively [7]. Our patient had a hospital floor stay of one day and an ICU stay of two days, emphasizing his rapid decompensation. Based on this study, acute respiratory distress syndrome (ARDS) was seen in 34%, renal dysfunction was seen in 55%, hepatic dysfunction was seen in 64%, and coagulopathy developed in 69% of the patients. Additionally, 81% of the ICU patients with streptococcal toxic shock syndrome were intubated with mechanical ventilation, and 21% needed renal replacement therapy [7]. Our patient had all the previously mentioned conditions from the study [7], requiring a higher complexity of care with multidisciplinary involvement in stabilizing the patient in the ICU.

According to the current UpToDate guidelines, streptococcal toxic shock syndrome should be managed with the treatment of septic shock and its complications, surgical debridement of infection if applicable, antimicrobial therapy, and administration of IVIG. The antimicrobial regimen included empiric treatment for suspected streptococcal toxic shock syndrome and tailored treatment once the diagnosis of streptococcal toxic shock syndrome has been confirmed [8].

The empiric antibiotic regimen recommendation awaiting culture results includes clindamycin plus vancomycin plus a carbapenem or a combination of drugs containing penicillin plus a beta-lactamase inhibitor [8]. For individuals with known hypersensitivity to penicillin, clindamycin plus vancomycin plus a carbapenem is recommended for empiric treatment [8]. The tailored antibiotic regimen recommendation once cultures have been confirmed includes clindamycin plus penicillin G and should be continued with confirmed cultures susceptible to clindamycin [8]. If found to be resistant to clindamycin, penicillin plus linezolid is recommended [8]. Our patient was started on clindamycin, vancomycin, and zosyn after decompensating on ICU Day 1, pending blood culture results. He was then switched to meropenem, daptomycin, and clindamycin upon confirmation of group A Streptococcus bacteremia and started IVIG therapy on ICU Day 2.

A five-year retrospective study of 57 patients done by Fernandez-Galilea et al. across nine ICU facilities in Southern Spain showed that clindamycin use significantly reduced mortality in critically ill ICU bacteremic group A Streptococcus-infected patients after controlling for confounders. Additionally, this study indicated that IVIG was not a protective factor for ICU mortality [9]. Another study of 1,079 inpatients with invasive group A Streptococcus (iGAS) infection in comparison to 877 inpatients with invasive non-group A/B streptococcal (iNABS) infection in the United States showed that the use of clindamycin for iGAS infections improved in-hospital mortality outcomes compared to those who did not receive clindamycin [10]. In the case of our patient, the clindamycin use during treatment did not reduce his hospital mortality as he passed away on his second ICU day.

A study of 84 cases of severe group A Streptococcus infection in Australia done by Carapetis et al. showed that the reduced mortality effect of clindamycin treatment in the affected individuals may be enhanced by IVIG [11]. A study of 67 cases of invasive group A Streptococcus infection done by Linner et al. in Sweden showed a remarkably improved survival in patients who had both IVIG and clindamycin therapy [12]. However, the study by Mehta et al. on the morbidity and mortality of 62 ICU patients with streptococcal toxic shock syndrome across four university hospitals in Toronto, Canada, showed no relationship between IVIG use, surgery, or clindamycin and survival. The study indicated that patient mortality was directly linked with acute physiology, multiple comorbidities, and the amount of dysfunctional organs [7]. The findings of the study by Mehta et al. [7] are reflective of our patient as his mortality was directly correlated with the number of dysfunctional organs, multiple comorbidities, and rapid decompensation.

## Conclusions

Toxic shock syndrome can escalate to a life-threatening condition very rapidly. It classically presents with fever, hypotension, sunburn-like rash, and multiorgan system failure. This patient had an atypical presentation of this condition with left chest wall pain and left arm pain after two mechanical falls with hemodynamic stability. Following his second visit to the ED later that day for worsening pain, he was admitted for pain management and further imaging workup. During the admission stay, he rapidly decompensated, resulting in the escalation of care to the ICU and passed away on his second ICU day. His hospital admission consisted of a hospital floor stay of one day and an ICU stay of two days, where he passed away on the second ICU day, emphasizing his rapid decompensation. The aim of this case report is to illuminate the distinctive and complex manifestation of this patient's case, in order to assist in the diagnosis, handling, and therapy of this condition.
